# Electrical and Thermal Transport in CaCO_3_ Graphene Nanocomposites Derived from Oyster Shell Waste

**DOI:** 10.1021/acsomega.6c09705

**Published:** 2026-10-21

**Authors:** Chiara Marchini, Giulia Fredi, Stefano Goffredo, Alessandro Gradone, Andrea Migliori, Simona Fermani, Giuseppe Falini, Devis Montroni

**Affiliations:** † Department of Chemistry “Giacomo Ciamician”, University of Bologna, Via Piero Gobetti 85, 40129 Bologna, Italy; ‡ Department of Industrial Engineering and INSTM Research Unit, 19034 University of Trento, Via Sommarive 9, 38123 Trento, Italy; § Marine Science Group, Department of Biological, Geological and Environmental Sciences, University of Bologna, Via F. Selmi 3, 40126 Bologna, Italy; ∥ Fano Marine Center, The Inter-Institute Center for Research on Marine Biodiversity, Resources and Biotechnologies, Viale Adriatico 1/N, 61032 Fano, Italy; ⊥ Consiglio Nazionale delle Ricerche, Istituto per lo Studio dei Materiali Nanostrutturati (CNR-ISMN), Via P. Gobetti 101, 40129 Bologna, Italy; # Interdepartmental Centre for Industrial Research Health Sciences & Technologies, University of Bologna, 40064 Bologna, Italy

## Abstract

Waste oyster shells provide a calcium carbonate, as Mg-calcite, feedstock for functional mineral composites. Here, shell-derived biogenic calcium carbonate (BCC) and few-layer graphene (FLG; nominally 1, 5, 7, and 10 wt % relative to BCC) were processed by aqueous planetary ball milling with Pluronic P123. Microscopy, diffraction, spectroscopy, thermogravimetry, and transport measurements were combined to examine the resulting powders and pressed pellets. Mg-calcite nanocrystallites and graphene sheets coexisted within micrometer-scale aggregates, with mineral particles preferentially observed near sheet edges. At 25 °C, the thermal conductivity of the 10 wt % formulation was 0.516 W·m^–1^·K^–1^, compared with 0.194 W·m^–1^·K^–1^ for the dry-milled shell. This approximately 166% reference-to-composite difference combines changes in composition and processing and cannot be assigned to FLG addition alone. Stable electrical measurements were obtained from 5 wt % FLG; the reported resistivity of the 10 wt % formulation was approximately 0.50 Ω·cm. The shell organic matrix may contribute to interfacial interactions, although its effect cannot be distinguished from those of Pluronic and processing in the present experiments. The observed transport differences among the prepared formulations provide a basis for future studies using matched processing controls to clarify the contribution of each constituent.

## Graphical abstract

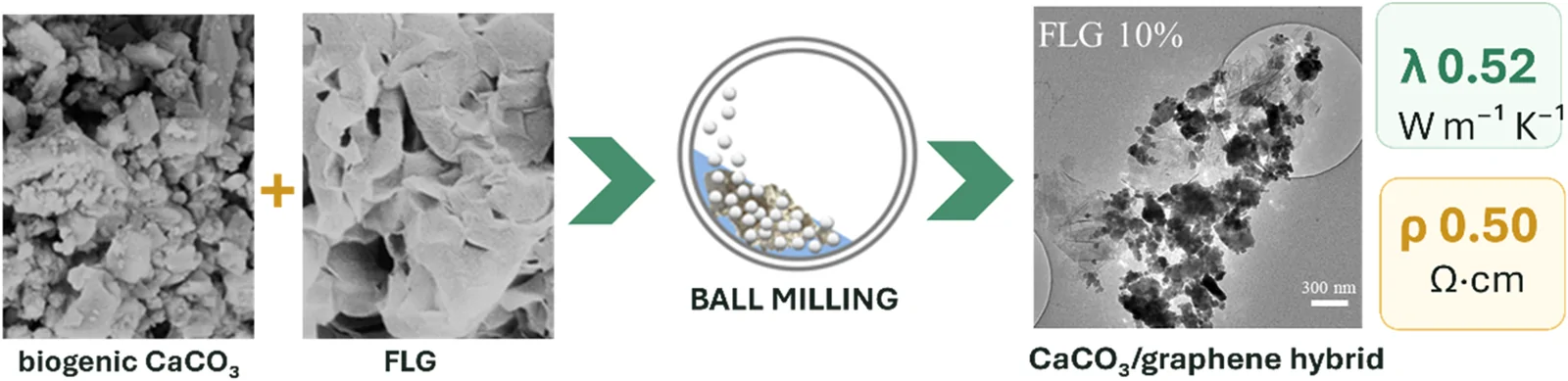

## Introduction

Nanocomposites combine constituents with nanoscale dimensions to modify properties such as mechanical response, barrier performance, or electrical and thermal transport. Calcium carbonate particles are established fillers in polymer composites, while conductive carbon fillers can introduce additional functionality.^1–4^ Their performance depends on the organization of the constituents and their interfaces, rather than on filler composition alone.

Calcium carbonate (CaCO_3_) is an abundant mineral and biomineral used in paper, polymers, construction, agriculture, and other industrial sectors.^5,6^ Its availability and established processing routes make it an attractive mineral component for composite development. However, CaCO_3_ is electrically insulating, and the heat transport of powder compacts is also affected by porosity and particle contacts. Incorporating conductive carbon offers a route to modify these effective properties.

Graphene and its derivatives have high electrical and thermal transport capabilities, although these depend on defects, layer structure, and measurement conditions.^7–9^ CaCO_3_ composites containing graphene oxide (GO) or reduced graphene oxide (rGO) have already been investigated, including their crystallization and structural characteristics.^10–12^ Mandal and Kumar reported ball-milled rGO/CaCO_3_ composites and structural, optical, and electrical-property interpretations in 2023; their optical band gap analysis should be distinguished from direct bulk resistivity measurements.^13^ Mandal et al. subsequently examined morphology, structure, thermal stability, and thermal transport in rGO/CaCO_3_ composites.^14^ Related application studies include a waste-oyster-shell/GO soil conditioner and a GO/nano-CaCO_3_ additive for cement mortar.^15,16^

The contribution of the present study is the preparation of a shell-derived Mg-calcite/FLG formulation series by aqueous milling and the joint examination of microstructure, composition, electrical resistivity, and thermal transport. It extends prior CaCO_3_/graphene work to an additional waste-derived feedstock and processing route.

Industrial CaCO_3_ is commonly obtained by grinding geogenic minerals or by precipitation, for example through carbonation of calcium hydroxide.^6,17^ Mollusk shells provide an alternative CaCO_3_-rich feedstock generated by seafood processing. Existing shell uses include liming, feed supplementation, and mineral fillers.^5,18–21^ Recent analysis of seafood-derived biominerals emphasizes that extraction and processing conditions determine both product characteristics and the environmental value of their recovery.^22^

This study uses biogenic calcium carbonate from *Crassostrea gigas* shells. Shell-derived BCC contains an organic matrix (OM) and structural features established during biomineralization, some of which can be modified by milling.^23,24^ Previous work has examined shell-derived adsorbents, covalent functionalization, and stearate coatings, showing that the organic and mineral fractions can influence surface modification in those systems.^23,25,26^ These findings motivate testing whether the OM also affects BCC/FLG interfaces; they do not demonstrate that it improves transport in the present composites.

The aim is to examine the electrical and thermal properties of BCC/FLG formulations and relate the observed trends to particle size, aggregation, and local mineral–graphene association. An OM-mediated interaction is considered as one possible contribution alongside Pluronic adsorption and milling-induced changes. Recent graphene-composite literature highlights the importance of interfacial resistance and network organization for heat transfer; the relevance of those mechanisms to BCC must be tested directly.^27^

The composite-forming step is carried out in water using Pluronic P123, without calcination of the shell-derived mineral. This approach provides a route for investigating waste valorization and formulation-associated transport properties. These results establish a basis for subsequent assessment of environmental performance, process scalability, and suitability for specific technological applications.

## Experimental Procedure

### Materials

Few-layer graphene (FLG) was supplied by GrapheneUP (Czech Republic). According to the supplier specifications, the material consisted of two to five stacked graphene layers, with an apparent thickness below 2.1 nm and an average lateral size of 20–50 μm. *Crassostrea gigas* shell powder was supplied by F.lli Terzi (Palosco, Italy). The shells were pretreated by washing with tap water, immersion in a 5 vol % sodium hypochlorite solution for 24 h, rinsing with deionized water, air-drying, and hammer milling. A 30 g portion of the resulting shell powder was subsequently dry-milled in a 500 mL zirconia jar containing 125 g of 20 mm zirconia balls using a Retsch PM 100 planetary ball mill (Retsch GmbH, Haan, Germany) operated at 400 rpm for 1 h. The milled powder was then passed through a 45 μm sieve. When more than 30 g of material was needed for an experiment multiple batches were mixed and homogenized. All characterizations were performed after homogenization. The starting material was characterized analyzing different batches in order to account for possible batch-to-batch variability. Pluronic P123 was used as a dispersing agent during aqueous FLG processing, while ethanol was employed for TEM specimen preparation. Milli-Q water (resistivity 18.2 MΩ·cm at 25 °C) was used for composite preparation. Unless otherwise specified, chemicals were purchased from Merck (Darmstadt, Germany) and used as received.

### Synthesis of the BCC/Graphene Nanocomposites

Before starting the nanocomposite preparation, the FLG was milled to increase the exfoliation and disaggregate the powder. For this purpose, 4.0 g of FLG, 50 mL of Milli-Q water, and 0.25 g of Pluronic P123 were added in the zirconia jar previously mentioned. Then, the dispersion was milled at 400 rpm in the planetary ball mill with 50 g of zirconia spheres (2 mm diameter) for 1 h.

The BCC/FLG composites were obtained by adding shell powder to the premilled FLG dispersion, followed by milling at 400 rpm for 6 h with 50 g of 2 mm zirconia balls. The resulting slurry was air-dried without removing a liquid fraction and sieved at 600 μm; no visible sieve residue was reported. The nominal loading x is interpreted here as mFLG/(mFLG + mBCC). On the literal basis of 4.0 g FLG in every batch, the BCC masses required for 1, 5, 7, and 10 wt % are 396.0, 76.0, 53.14, and 36.0 g, respectively. The corresponding ball-to-(BCC + FLG) mass ratios are 0.125, 0.625, 0.875, and 1.25. The dry-milled shell powder is used as a reference. No additional water was added during the milling process, except for the water introduced in with the FLG dispersion. As a result, the consistency of the milling mixture depended on the FLG concentration. In particular, at low FLG concentrations (e.g., 1%), the mixture exhibited a highly viscous, paste-like consistency, whereas at higher FLG concentrations (e.g., 10%), it formed a less viscous slurry.

### Morphological Characterization

Scanning electron microscopy (SEM) observations were performed on the dry synthesized BCC/graphene nanocomposites. Using a spatula, a small amount of sample was placed on the surface of a carbon tape attached to an aluminum stub, then the excess was removed using a gentle stream of air. The sample was then coated with a thin layer (<10 nm) of Au. The observations were conducted using a ZEISS Leo 1530 Gemini field emission gun scanning electron microscope (FEG-SEM) at a voltage of 5 kV, aperture of 20 μm, and a working distance of 7 mm.

High-resolution transmission electron microscopy (HR-TEM) images and electron diffraction patterns were collected using a FEI Tecnai F20ST TEM operated at 200 kV. Samples were prepared by suspending a small amount of powder in ethanol and depositing a drop of the suspension directly onto a 200-mesh Cu grid with a holey-type carbon film. The solvent was evaporated at 80 °C before analysis.

### Granulometry

The particle size distribution of the samples was measured using a Malvern Mastersizer 2000 laser diffraction particle size analyzer (Malvern Panalytical) with a measurement range of 0.02–2000 μm. The particles from each sample were dispersed in distilled water for the measurement.

### X-ray Powder Diffraction (XRPD)

XRPD patterns were collected using a PanAnalytical X’Pert Pro diffractometer with an X’Celerator detector and Cu Kα radiation generated at 40 kV and 40 mA (λ = 1.54056 Å). The reported scan range was 20–60° in 2θ, with a step size of 0.05° and a counting time of 60 s. Profex was used for phase identification, crystalline-phase quantification, and calcite lattice-parameter analysis. The crystallite-size measure in Table 1 is denoted *L*_104_, to distinguish it from the interplanar spacing d_104_. In a Scherrer analysis, *L*_104_ = *K*λ/(βsize cos θ_104_), where *K* is the shape factor, θ_104_ is half the peak position in 2θ, and βsize is the size-related peak breadth in radians of 2θ. Instrumental broadening was assumed to be negligible in the reported estimates; the *L*_104_ values are therefore treated as apparent coherent-domain sizes.

**Table 1:** Crystalline-Phase Fractions, Apparent Calcite Coherent-Domain Size Normal to (104), *L*_104_, and Laser-Scattering Particle-Size Percentiles^a^

Sample	Mg-calcite (wt %)	FLG (wt %)	*L*_104_ (nm)	*D*_(10)_ (μm)	*D*_*(*50)_ (μm)	*D*_(90)_ (μm)
oyster shell powder	100^b^	n.d.	117 ± 8	1.47 ± 0.03	16.9 ± 0.6	49.1 ± 0.7
FLG	n.d.	100^b^	NA	15 ± 1	35 ± 4	69 ± 7
FLG 1%	99.1 ± 0.3	0.9 ± 0.3	47 ± 3	0.74 ± 0.01	9.6 ± 0.3	56 ± 4
FLG 5%	97.4 ± 0.6	2.6 ± 0.6	42 ± 8	0.73 ± 0.07	17.9 ± 0.4	56 ± 2
FLG 7%	94 ± 3	6 ± 3	39 ± 6	0.9 ± 0.1	5.5 ± 0.8	47 ± 2
FLG 10%	92 ± 4	8 ± 4	45 ± 8	0.38 ± 0.01	7.2 ± 0.3	48 ± 2

a *L*_104_ is a domain-size estimate. Phase fractions refer to the modeled crystalline phases. *D*_(10)_, *D*_(50)_, and *D*_(90)_ were measured after dispersion in water.

b Single detected crystalline phase. n.d. = not detected; NA = not applicable.

### Spectroscopic Analyses

The Raman spectra were collected on the dry powder of the samples using a Renishaw Raman Invia spectrometer (Renishaw plc, United Kingdom) equipped with a Leica DMLM optical microscope. Two laser sources, both equipped with an edge filter to reduce Rayleigh scattering, were available: an Ar^+^ laser (514.5 nm, *P*_max_ = 30 mW) with an 1800 lines·mm^–1^ monochromator, and a diode laser (780.0 nm, *P*_max_ = 300 mW) with a 1200 lines·mm^–1^ monochromator. The detector used was a CCD (Charge-Coupled Device) thermoelectrically cooled at 203 K. The instrument was calibrated with a silicon wafer (520 cm^–1^). The laser was focused on the sample using a 50× magnification, a laser power of 1.5 mW, and for each sample four scans of 10 s each were acquired. The baseline correction was performed using the Renishaw WiRE 2.0 software. Three specimens were analyzed for each sample type. A high fluorescence was observed associated with the calcite phase and increased as the graphene content decreased.

The infrared spectra were collected using a Thermo Scientific Nicolet iS10 and processed with OMNIC software (Thermo Electron Corp., Woburn, MA). The dry samples were measured in ATR mode and the spectra were collected with 2 cm^–1^ resolution and 70 scans.

### Thermogravimetric Analysis (TGA)

The TGA was performed using an SDT Q600 V 8.0 instrument (TA Instruments). The system was pre-equilibrated at 30 °C, and then a ramp was performed from 30 to 900 °C with a 10 °C·min^–1^ heating rate under a nitrogen flow (100 mL·min^–1^). The measurements were performed on about 12 mg of sample each in an alumina crucible.

### Electrical Resistivity Measurements

Electrical measurements were performed using a Jandel Multi Height four-point probe, an AMEL model 2053 potentiostat/galvanostat for current supply, and a Hewlett-Packard 3478A multimeter for voltage measurement. Pellets of 20 mm diameter were compacted at 12 MPa for 1 min at room temperature. Thickness was measured with a micrometer (±0.005 mm), and three replicates per formulation were examined at 10 μA, 100 μA, and 1 mA. The original calculation used ρ = *RA*/*l*, with *R* the measured resistance, *A* the pellet cross-sectional area, and *l* its thickness. This expression assumes a uniform current path through that cross-section and is not the general conversion for a collinear surface four-point probe.

### Thermal Conductivity Measurements

Thermal diffusivity (α) was measured at 25 °C using an LFA 467 light flash analyzer (Netzsch, Selb, Germany). Nominally 12.8 mm diameter specimens were prepared by compressing 0.5 g of powder in a steel mold at 128 MPa for 1 min at room temperature using a Carver press. Three specimens per formulation were measured, with five pulses per specimen. Both faces were coated with graphite spray to increase absorption and emissivity. Data were fitted in Proteus using the reported standard model, linear baseline, and exponential pulse correction. Specific heat capacity (*C*_p_) was determined by comparison with Pyroceram 9606. Geometric density (*D*) was calculated from specimen mass and cylindrical volume; balance, caliper, and micrometer resolutions were 0.0001 g, 0.01 mm, and 0.001 mm, respectively. Thermal conductivity was calculated as λ = α*C*_p_ *D*. With α in mm^2^·s^–1^, *C*_p_ in J·g^–1^·K^–1^, and *D* in g·cm^–3^, the numerical product gives λ in W·m^–1^·K^–1^. Thermal and electrical measurements used different compaction pressures and therefore do not probe identical pellet microstructures.

### Statistical Analyses

Statistical analyses of the electrical resistivity and thermal conductivity data were performed using the R programming language in the RStudio environment. Data normality was assessed using the Kolmogorov–Smirnov test, while homogeneity of variance was evaluated using Levene’s test. Given the limited sample size (*n* = 3 per group), the results of these tests were interpreted with caution and were considered together with the observed distribution and variability of the data. When the assumptions for parametric analysis were considered adequately supported, differences among groups were assessed using one-way analysis of variance (ANOVA), followed by Tukey’s honestly significant difference (HSD) test for post hoc pairwise comparisons in the case of significant ANOVA results. When the assumptions for parametric analysis were not considered adequately supported, the nonparametric Kruskal–Wallis test was used instead, followed by Dunn’s test for post hoc pairwise comparisons.

Based on this assessment, thermal conductivity data did not meet the normality assumption and were therefore analyzed using the Kruskal–Wallis test followed by Dunn’s post hoc test. In contrast, electrical resistivity data met both the normality and homogeneity of variance assumptions and were analyzed using one-way ANOVA followed by Tukey’s HSD post hoc test.

## Results and Discussion

### Particle Size Distribution

To prepare a nanocomposite material of biogenic calcite and graphene, the two starting materials, oyster CaCO_3_ powder and the FLG, were both initially milled to reduce their particle size. While the oyster shell was milled in dry conditions, the FLG was milled in a wet environment in the presence of a surfactant (Pluronic) to enhance the graphene exfoliation. The SEM images of the dry powders of the milled starting materials shown in Figure 1 show particles ranging from <1 μm up to tens of microns for the oyster powder and a highly aggregated morphology for the FLG, with aggregates of tens of microns and no clearly visible single graphene layers. This last morphology is likely due to a reaggregation of the graphene upon drying. These observations are confirmed by laser granulometry analyses (Table 1 and Figure S1) where the oyster powder shows 80% of the particle size distribution between 1 and 50 μm whereas for FLG it lies between 15 and 70 μm.

**Figure 1:**
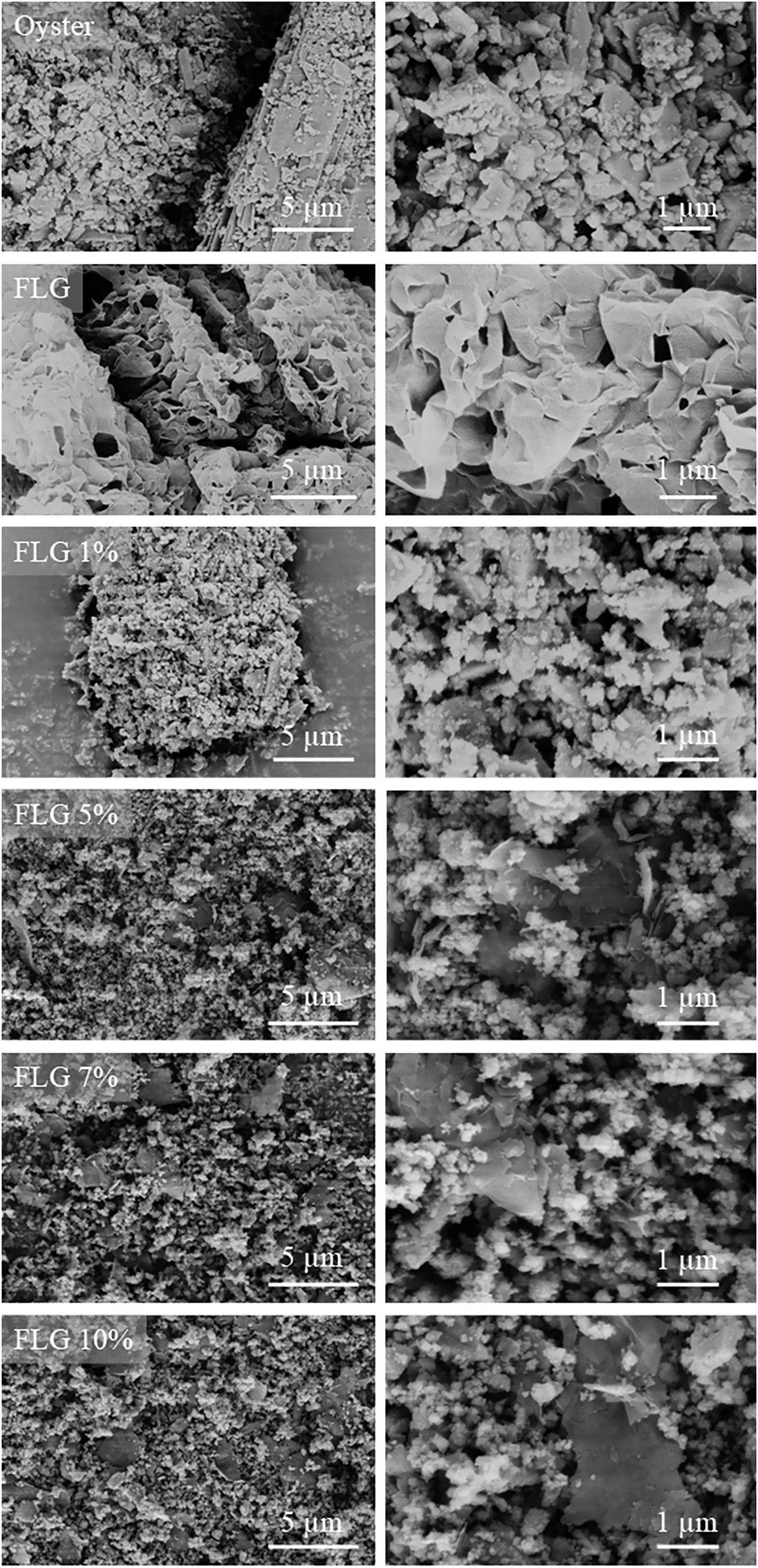
SEM images of the two starting materials, oyster shell powder and FLG, and of the four nanocomposite powders, FLG 1%, FLG 5%, FLG 7%, and FLG 10%. On the left, micrographs collected with a 15 k× magnification, on the right, micrographs collected with a 50 k× magnification.

The two starting materials were then mixed and wet milled together and air-dried to obtain the final nanocomposite powders. The SEM observation of the composites showed their tendency to aggregate into clusters ranging from about 1 μm to tens of microns. When observed at higher magnification, these clusters showed a fine structure where mostly nanoparticles are present. Single sheets of graphene, with nanometric thickness and few microns wide, are observed embedded into these nanoparticle-based matrix. Increasing the graphene content, a higher number of sheets are observed.

Granulometry measurements (Table 1 and Figure S1) were consistent with the SEM observations but gave a broader picture of these aggregate distributions. The data suggest that increasing the amount of FLG in the sample mildly decreases the particle size of the composites, giving similar results for FLG 7% and FLG 10%.

For FLG 5%, the slurry before drying had *D*_(10)_ = 0.30 ± 0.07 μm, *D*_(50)_ = 1.2 ± 0.5 μm, and *D*_(90)_ = 5.5 ± 0.9 μm. After air-drying and redispersion for granulometry, the corresponding median and upper percentile were 17.9 ± 0.4 and 56 ± 2 μm (Table 1). These results indicate pronounced aggregation during drying. Although the wet-slurry particle-size distribution remains predominantly within the submicrometer-to-micrometer range, complementary microscopy and diffraction analyses confirm the presence of primary nanoscale mineral domains, highlighting the hierarchical organization of the material across multiple length scales.

Mandal et al. used a different rGO/CaCO_3_ preparation involving 24 h ball milling, isopropyl alcohol washing, and vacuum drying.^14^ Accordingly, the observed differences in particle size are more appropriately interpreted in the context of the distinct starting materials and processing conditions rather than attributed solely to the biogenic origin of the CaCO_3_.

Although milling speed and duration were kept constant, the ball-to-solid and liquid-to-solid ratios varied among formulations. These differences could affect the mechanical exposure of the solids and the rheology of the milling mixture. Transport differences may reflect the combined effects of formulation and processing, thus a comparative series with matched ball-to-solid and liquid-to-solid ratios, jar filling, and milling schedule would help clarify their respective contributions. However, the observed small variations in particle size, *L*_104_, and Raman *I*_(D)_/*I*_(G)_ show no clear dependence on these processing differences.

Aggregation may create some local conductive contacts; the net effect depends on connectivity across the pellet. Controlled drying has been shown to influence sheet organization and heat transfer in graphene-based films, although that architecture differs from the present mineral powder.^28,29^

### Chemical and Crystallographic Characterization

XRPD identified Mg-calcite as the only detected CaCO_3_ phase in the starting shell powder and composites (Figure 2 and Table S1).^30^ The graphitic (002) reflection became more apparent with increasing FLG loading. Small changes in calcite lattice parameters were also observed. Previous studies have linked intracrystalline organics to lattice distortions in biogenic carbonates.^31–33^

**Figure 2:**
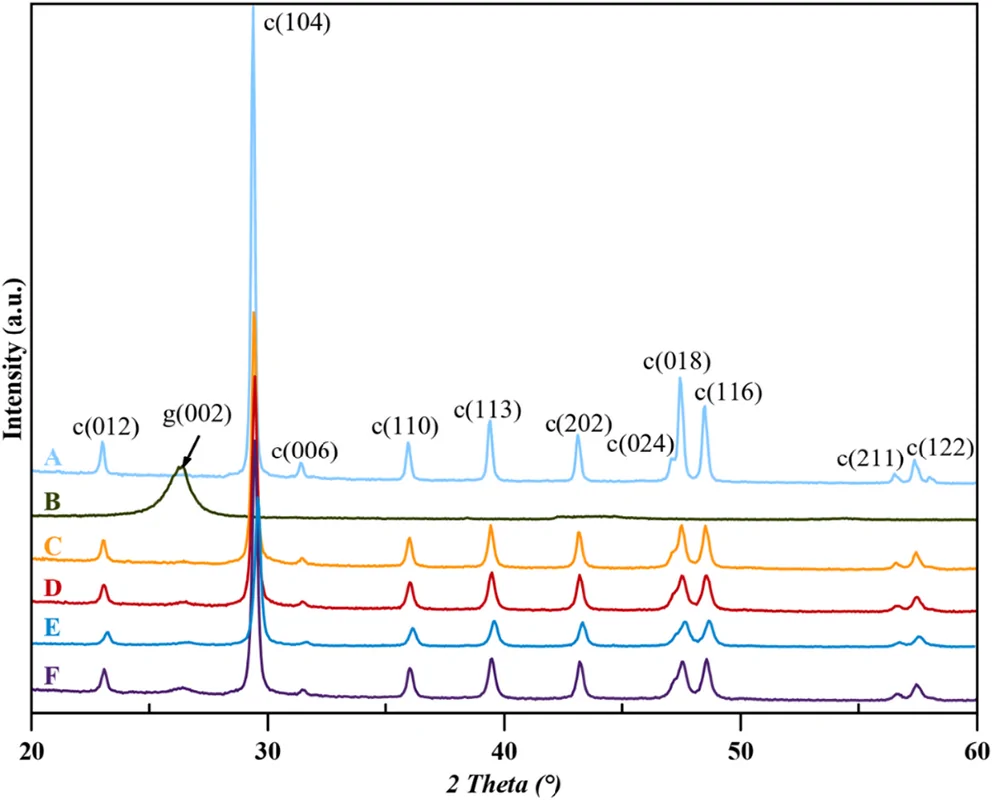
X-ray powder diffraction patterns of (A) oyster shell powder, (B) FLG, (C) FLG 1%, (D) FLG 5%, (E) FLG 7%, and (F) FLG 10%. c, calcite diffraction peak; g, graphene diffraction peak.

The reported *L*_104_ values decrease from 117 ± 8 nm in the shell reference to approximately 39–47 nm in the composites. This trend is consistent with milling-related reduction of coherent mineral domains and with the microscopy observations.^24,34^

FTIR spectra (Figure S2) were dominated by carbonate bands. Relative to the shell reference, the composites showed the reported ν_2_ shift from 871 to 874 cm^–1^ and ν_3_ shift from 1399 to 1418 cm^–1^, while ν_4_ remained near 712 cm^–1^. These changes indicate altered vibrational environments.^35–38^ Raman spectra showed carbonate stretching near 1085–1087 cm^–1^ and a lattice mode near 274–283 cm^–1^ (Figure 3 and Table 2).^39–41^ The lattice-mode change may reflect size, strain, or local structural differences.

**Figure 3:**
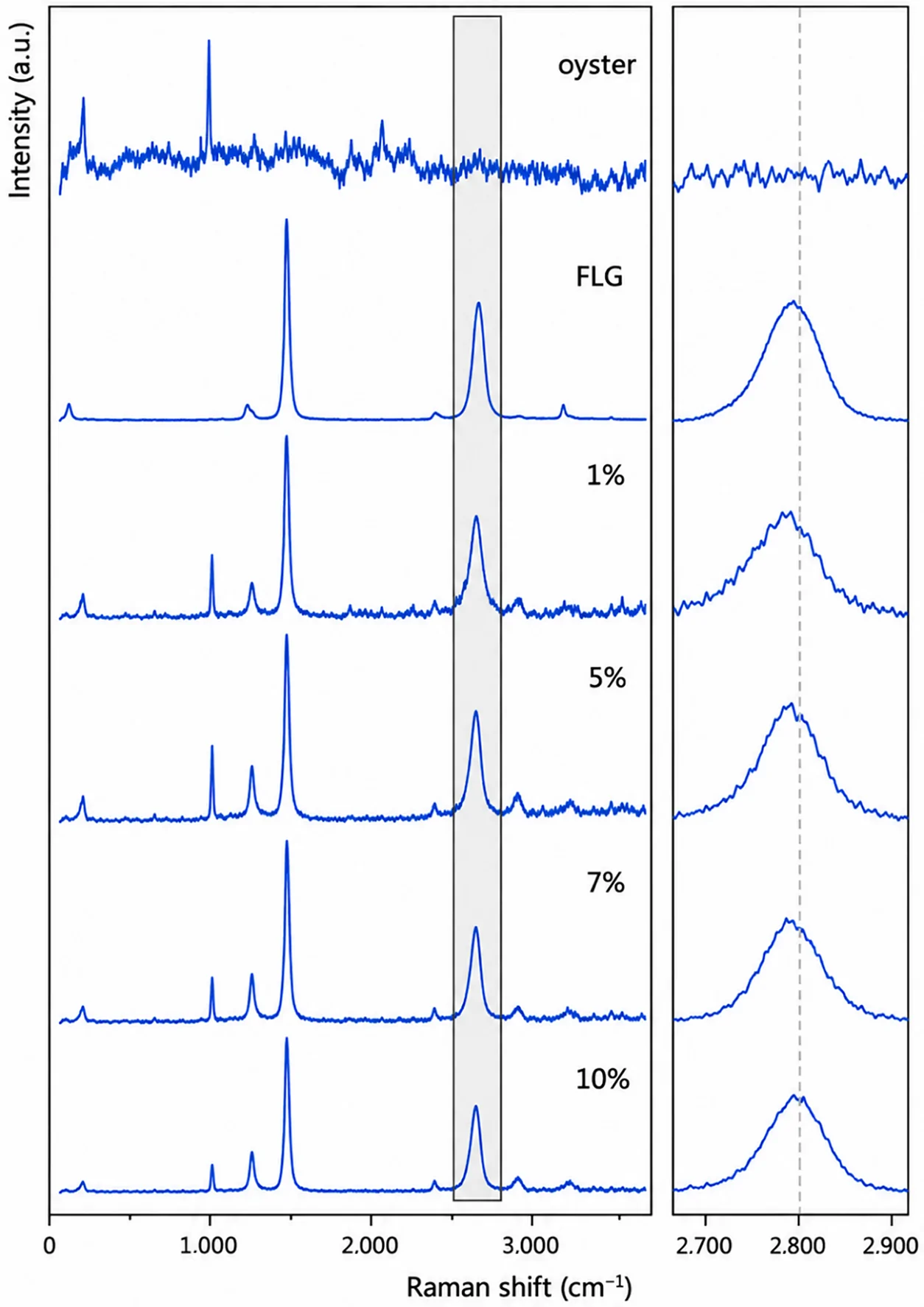
Raman spectra of BCC/FLG composites and an expanded view of the graphene G′ (2D) band. The spectra were processed using the reported 9-point smoothing procedure.

**Table 2:** Raman Shift (cm^–1^) of the Sample Powders^a^

	Graphene (FLG)					Mg-calcite	
	2D′ band	G′ band	G band	D band	*I*_(D)_/*I*_(G)_	CO_3_^2–^ symmetric stretching	Lattice mode
oyster shell	n.d.	n.d.	n.d.	n.d.	n.a.	1087 ± 1	283 ± 1
FLG	3247 ± 1	2707 ± 1	1582 ± 1	1352 ± 2	0.08 ± 0.01	n.d.	n.d.
FLG 1%	n.d.	2695 ± 1	1573 ± 1	1349 ± 1	0.21 ± 0.08	1086 ± 1	278 ± 2
FLG 5%	n.d.	2709 ± 2	1581 ± 1	1353 ± 2	0.29 ± 0.01	1086 ± 1	276 ± 1
FLG 7%	3248 ± 4	2708 ± 2	1580 ± 1	1354 ± 1	0.26 ± 0.02	1085 ± 1	274 ± 3
FLG 10%	3248 ± 3	2707 ± 4	1579 ± 3	1350 ± 1	0.30 ± 0.02	1086 ± 1	276 ± 1

a The data are reported as average ± error (the highest between standard deviation and spectral resolution). Peak attribution was done according to literature data.^40–43^ n.d. = not detected. n.a. = not applicable.

The graphene Raman bands are reported in Table 2. The G and G′ bands of FLG 1% occur at lower wavenumbers than those of the other formulations (see Figure 3). Layer number, stacking, strain, doping, defects, and excitation wavelength can all influence these bands.^42–46^ The shifts are compatible with changes in graphene structure. The *I*_(D)_/*I*_(G)_ ratio increased from 0.08 ± 0.01 in the starting FLG to 0.21–0.30 in the composites (Table 2). This indicates a change in Raman-active disorder or edge contributions under the measurement conditions.^43,45,47^ Fragmentation during milling can increase the relative contribution of sheet edges, while additional basal-plane defects may also contribute.

### Nanoscale characterization

TEM analysis revealed mixed clusters of Mg-calcite particles and graphene sheets in the deposited specimens (Figure 4), with mineral particles frequently localized near sheet edges. This observation supports the presence of local mineral–graphene associations and suggests that defect-rich edge regions may contribute to interfacial interactions. At the same time, the observed organization may also reflect contributions from surface chemistry, Pluronic adsorption, and rearrangement during drying.

**Figure 4:**
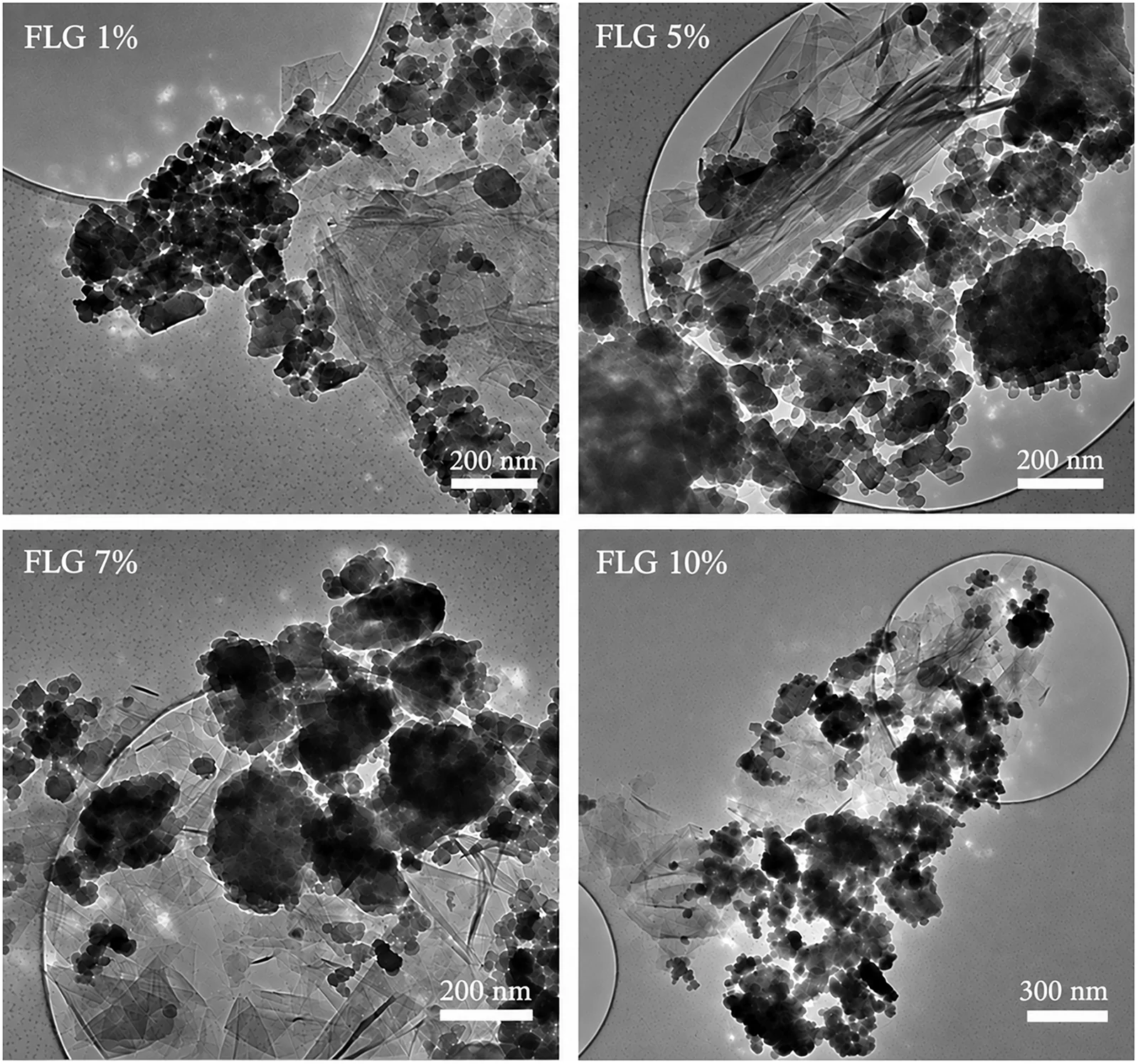
TEM micrographs of FLG 1%, FLG 5%, FLG 7%, and FLG 10%, showing aggregates composed of Mg-calcite nanoparticles and graphene sheets.

The preferential association of mineral particles with graphene-sheet edges may influence transport through several complementary mechanisms. By limiting complete face-to-face restacking, Mg-calcite particles may promote a more distributed organization of the graphene sheets and preserve basal-plane regions available for intersheet contacts. Conversely, electrically insulating mineral particles located at sheet junctions may increase separation between conductive domains, thereby increasing tunneling distances and interfacial contact resistance and potentially delaying the formation of a continuous electrical network. For thermal transport, mineral contacts may locally bridge adjacent regions, while the increased number of mineral–graphene interfaces can also enhance phonon scattering and interfacial thermal resistance. Thus, transport is expected to depend on the balance between network continuity and interfacial resistance, both recognized as key design parameters in graphene-based composites.^27^ These observations are consistent with the pronounced decrease in electrical resistivity between 5 and 10 wt % FLG and the more gradual enhancement in thermal conductivity.

The Mg-calcite particles in all composites showed a heterogeneous size distribution, ranging from approximately 15–20 to 100–300 nm. Compared with the starting oyster-shell powder (Figure S3), the composites contained smaller mineral particles. Consistent with SEM observations, many of the larger features appeared to be aggregates of smaller particles; this morphology was less apparent in the starting powder.

FFT analyses of high-resolution micrographs (Figure S4) showed spacings reported as 0.27, 0.24, and 0.17 nm, compatible with calcite-family domains, together with features near 0.21 and 0.34 nm potentially attributable to graphitic domains. The 0.34 nm feature is compatible with graphitic (002).

### Thermal and Electrical Behavior

TGA was performed under the air atmosphere. The reported mass-loss events and derivative peak temperatures are shown in Table 3. Carbonate decomposition dominates the high-temperature loss of shell-containing samples. Neat FLG exhibited a total weight loss of approximately 14 wt % from 30 to 900 °C, leaving about 86 wt % residue (Figure S5). This weight loss started at about 470 °C and was not associated with a distinct thermal event. Considering specifically the temperature range corresponding to calcium carbonate decarbonation (540–800 °C), approximately 8 wt % of the total weight loss was observed within this interval.

**Table 3:** Reported TGA Mass Losses and DTG Peak Temperatures^a^

	Dehydration	Organic matrix weight loss (150–450 °C)		High-temperature event (∼540–800 °C)	
Sample	weight loss (wt %)	*T*_max_ (°C)	weight loss (wt %)	*T*_max_ (°C)	weight loss (wt %)
oyster shell	0.35	∼290	1.50	734	42.10
FLG	0.45	n.d.	∼0.6	n.d.	∼8
FLG 1%	0.59	290	1.72	739	42.38
FLG 5%	0.67	302	2.15	747	40.36
FLG 7%	0.66	306	2.11	751	40.38
FLG 10%	0.55	315	2.89	751	39.69

a The intermediate-temperature loss is an operational 150–450 °C interval for organic material measurement. The high-temperature weight loss measured approximately within the 540–800 °C range, depending on the actual temperature range of the corresponding thermal event, is primarily associated with the decarbonation of calcium carbonate, with a smaller contribution from the progressive, non-specific thermal degradation of graphene.

All samples showed a small reported loss below 150 °C, approximately 0.3–0.7 wt %, consistent with removal of adsorbed moisture. The 150–450 °C interval can include degradation of shell OM and Pluronic, with possible contributions from other volatile species or carbon-surface groups.^23,24,48–53^ The shell reference showed a 1.50 wt % loss, while the composites showed 1.72–2.89 wt % and apparent DTG peak temperatures of 290–315 °C. These interval losses cannot be used to determine the shell organic-matrix content independently of Pluronic.

OM released or exposed during milling could adsorb on graphene and modify local contacts without changing total OM content. Biomolecule–graphene adducts have influenced calcite growth in a different model system.^54^ In the present composites, this mechanism is proposed as a plausible working hypothesis, with Pluronic also considered as a potential contributor to comparable interfacial effects.

The high-temperature DTG maximum increased from 734 °C for the shell reference to 751 °C for FLG 7% and FLG 10%. This is an apparent shift of a composite decomposition/oxidation event under the specified heating program. It is compatible with altered decomposition kinetics or gas transport.

Thermal properties were measured on pressed specimens; geometric density D, specific heat capacity *C*_p_, and thermal diffusivity α were combined to obtain λ. The reported values are given in Table 4 and Figure 5A,B; additional thermal results are referenced in Figure S7. Formulation means are described below.

**Figure 5:**
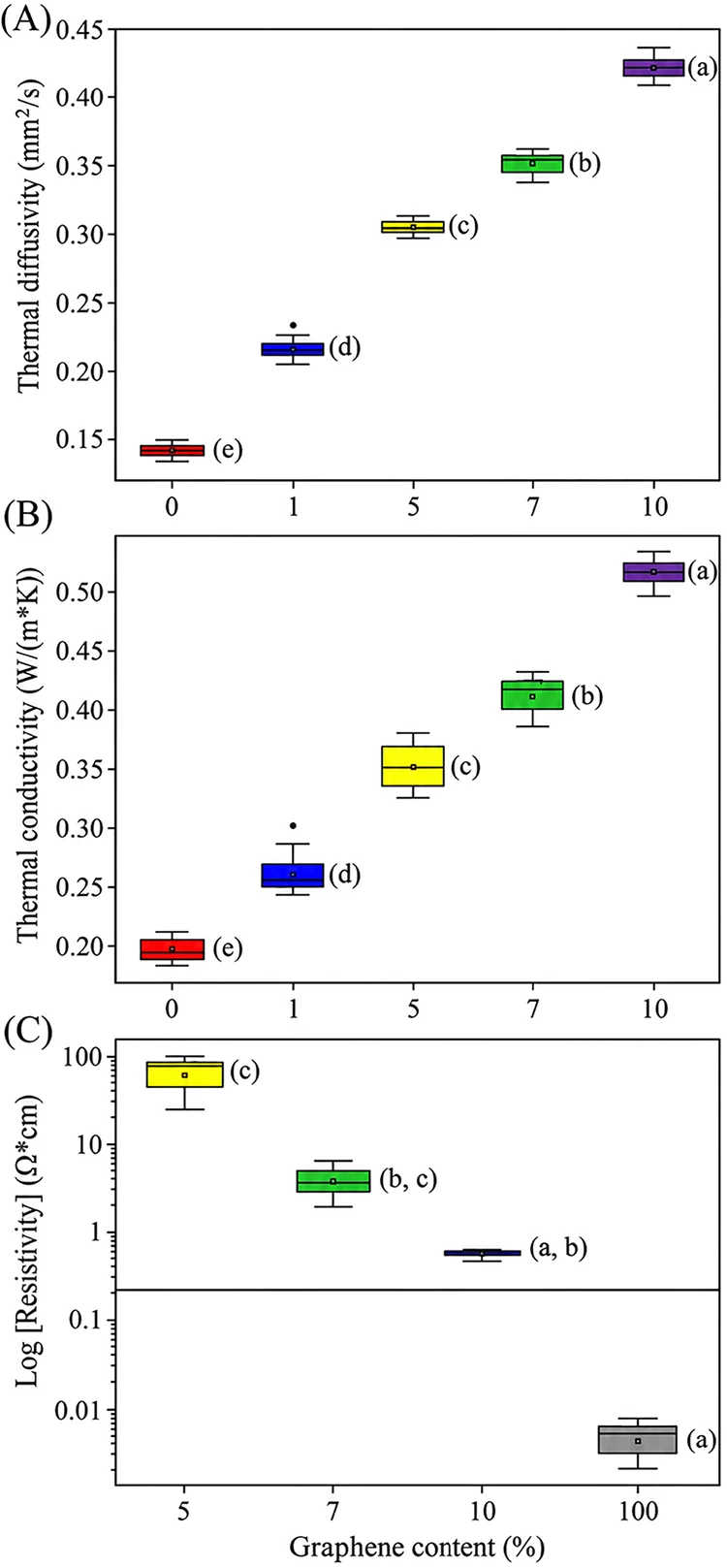
Original plots of thermal diffusivity (A), thermal conductivity (B), and electrical resistivity at 10 μA on a logarithmic scale (C). The 0 wt % label identifies the reference; 100 wt % identifies neat FLG. Letters within each graph represent the results of the statistical analysis (same letter indicates no significant difference).

**Table 4:** Geometric Density *D*, Thermal Diffusivity α, Specific Heat Capacity *C*_p_, and Thermal Conductivity λ of Pressed Specimens at 25 °C^a^

Sample	*D* (g·cm^‑3^)	α (mm^2^·s^‑1^)	*C*_p_ (J·g^‑1^·K^‑1^)	λ (W·m^‑1^·K^‑1^)
oyster shell	1.789 ± 0.004	0.142 ± 0.004	0.76 ± 0.03	0.194 ± 0.009
FLG 1%	1.554 ± 0.013	0.216 ± 0.006	0.77 ± 0.04	0.259 ± 0.014
FLG 5%	1.462 ± 0.020	0.306 ± 0.004	0.77 ± 0.05	0.346 ± 0.017
FLG 7%	1.471 ± 0.018	0.353 ± 0.007	0.78 ± 0.04	0.406 ± 0.014
FLG 10%	1.503 ± 0.006	0.423 ± 0.007	0.81 ± 0.02	0.516 ± 0.011

a Three specimens per formulation were used.

The measured density decreased from 1.789 g·cm^–3^ for the shell reference to 1.462–1.554 g·cm^–3^ for the composites (Table 4). Thus, the higher mean thermal conductivity is not accompanied by an increase in geometric density. Geometric density alone does not distinguish changes in constituent density from changes in porosity or particle contacts.

The reported mean *C*_p_ values ranged from 0.762 to 0.810 J·g^–1^·K^–1^. Their limited variation is compatible with the heat capacities of the major constituents.^55,56^

Thermal diffusivity increased from 0.142 mm^2^·s^–1^ for the shell reference to 0.423 mm^2^·s^–1^ for FLG 10%. The four composite means increase approximately linearly over 1–10 wt % FLG.

Thermal conductivity increased from 0.194 W·m^–1^·K^–1^ for the dry-milled shell reference to 0.259, 0.346, 0.406, and 0.516 W·m^–1^·K^–1^ for FLG 1%, FLG 5%, FLG 7%, and FLG 10%, respectively. Relative changes calculated as 100­(λ_sample_/λ_reference_ – 1) are approximately 34, 78, 109, and 166%. These are reference-to-composite differences that combine FLG/Pluronic addition with changes in milling, drying, aggregation, and pellet microstructure; they do not quantify an isolated FLG contribution. The observed trend is consistent with an increasing contribution from conductive-phase connectivity, while the specific microscopic mechanism remains to be established. The 1 wt % formulation therefore exhibits a higher mean thermal conductivity than the shell reference, indicating an early improvement in heat transport.

Mandal et al. reported an approximately 35% thermal-conductivity increase at 5 wt % rGO relative to their own CaCO_3_ reference.^14^ The approximately 78% reference-to-composite difference for our FLG 5% formulation also includes processing differences. The reported electrical values are listed in Table 5 and plotted in Figure 5C. No reliable value was obtained for the shell reference. Stable measurements were carried out for FLG 5%, FLG 7%, and FLG 10%. At 10 μA, their mean resistivities were approximately 60, 3, and 0.49 Ω·cm, respectively.

**Table 5:** Reported Electrical Resistivity at Three Applied Currents, Expressed as Mean ± Standard Error for Three Replicates^a^

	Electrical Resistivity (Ω·cm)		
Sample	at 10 μA	at 100 μA	at 1 mA
oyster shell	n.d.	n.d.	n.d.
FLG	0.006 ± 0.002	0.004 ± 0.001	0.005 ± 0.001
FLG 1%[Table-fn t5fn2]	∼70,000	n.d.	n.d.
FLG 5%	60 ± 20	60 ± 20	60 ± 20
FLG 7%	3 ± 1	4 ± 1	4 ± 1
FLG 10%	0.49 ± 0.04	0.50 ± 0.03	0.51 ± 0.03

a n.d. denotes no reliable value within the measurement conditions.

B No accurate or stable measurement was possible due to the high sample resistivity.

The mean resistivity of FLG 10% at 10 μA (0.49 Ω·cm) is approximately 82 times the neat-FLG mean (0.006 Ω·cm). The corresponding ratios at 100 μA and 1 mA are approximately 125 and 102.

Jin et al. examined graphene/cement composites by alternating-current methods, illustrating the strong dependence of electrical response on conductive pathways in a different mineral matrix.^57^ Such results provide qualitative context, not a direct quantitative benchmark for the present compacted powders. In this study, the transition from an unstable 1 wt % response to stable 5 wt % measurement identifies an operational interval for measurable conduction under the stated conditions.

## Conclusion

Aqueous ball milling produced oyster-shell-derived Mg-calcite/FLG composites containing nanoscale mineral domains and graphene within larger aggregates. Their combined morphological, structural, compositional, electrical, and thermal characterization extends the investigation of CaCO_3_/graphene materials to a shell-derived feedstock and the reported water-based preparation.

At 25 °C, the reported thermal conductivity reached 0.516 W·m^–1^·K^–1^ for FLG 10%, compared with 0.194 W·m^–1^·K^–1^ for the dry-milled shell reference. The approximately 166% difference is a reference-to-composite comparison that combines composition and processing changes; it cannot be attributed to FLG addition alone. As no structural differences were observed using XRD and Raman spectroscopies among samples with different FLG concentration, it is a reasonable assumption that the effect is FLG driven. Stable electrical measurements were reported from 5 wt % FLG, and FLG 10% had the lowest reported composite resistivity, approximately 0.50 Ω·cm.

The measured properties support evaluating these powders as multifunctional fillers in a binder or matrix. The reported resistivity of the 10 wt % compact corresponds nominally to about 2 S·cm^–1^ (200 S·m^–1^). The measured thermal conductivity of 0.516 W·m^–1^·K^–1^ demonstrates a meaningful enhancement in heat transport within the compacted composite and provides a promising basis for applications requiring moderate thermal management. Table S3 relates plausible uses to the evidence available.

## Supplementary Material



## Notes

Particle-size distributions (Figure S1); FTIR spectra (Figure S2); TEM images and FFT analyses (Figures S3 and S4); TGA/DTG results (Figures S5 and S6); additional thermal and statistical results (Figure S7); Mg-calcite diffraction parameters (Table S1); component balance for the 150–450 °C loss (Table S2); and prospective uses of the shell-derived BCC/FLG powder (Table S3) (PDF)
