# Supplementary material for: Electrical and Thermal Transport in CaCO3 Graphene Nanocomposites Derived from Oyster Shell Waste
Source: ACS Omega. 2026 Oct 21;11(43):67843–52. doi: 10.1021/acsomega.6c09705 (PMC13737975; doi:10.1021/acsomega.6c09705)
Supplement: Supplementary file 1 [file ao6c09705_si_001.pdf]

SUPPORTING INFORMATION

Electrical and Thermal Transport in $CaCO_3$ Graphene Nanocomposites Derived from Oyster Shell Waste

Chiara Marchini[1], Giulia Fredi[2], Stefano Goffredo,[3,4] Alessandro Gradone[5], Andrea Migliori[5], Simona Fermani [1,6], Giuseppe Falini[1], and Devis Montroni[1,*]

[1] Department of Chemistry "Giacomo Ciamician", University of Bologna, Via Piero Gobetti 85, 40129 Bologna, Italy.

[2] Department of Industrial Engineering and INSTM Research Unit, University of Trento, Via Sommarive 9, 38123, Trento, Italy.

[3] Marine Science Group, Department of Biological, Geological and Environmental Sciences, University of Bologna, Via F. Selmi 3, 40126 Bologna, Italy.

[4] Fano Marine Center, The Inter-Institute Center for Research on Marine Biodiversity, Resources and Biotechnologies, Viale Adriatico 1/N, 61032 Fano, Italy.

[5] Consiglio Nazionale delle Ricerche, Istituto per lo Studio dei Materiali Nanostrutturati (CNR-ISMN), Via P. Gobetti 101, 40129 Bologna, Italy.

[6] Interdepartmental Centre for Industrial Research Health Sciences & Technologies, University of Bologna, 40064 Bologna, Italy.

Figure S1. Particle size distribution profiles of the laser granulometric analyses. p. 2

Figure S2. FTIR spectra of nanocomposites and starting materials. p. 3

Figure S3. TEM micrographs at different magnification of the oyster shell powder. p. 4

Figure S4. High-Resolution TEM of the nanocomposites. p. 5

Figure S5. TGA profile of nanocomposites and starting materials. p. 6

Figure S6. TGA data on the organic matrix degradation. p. 7

Figure S7. Boxplots of the geometrical density and specific heat capacity. p. 8

Table S1. List of the main diffraction peak angles of calcite. p. 9

Table S2. Component balance for the 150–450 °C loss. p. 10

Table S3. Prospective uses of the shell-derived BCC/FLG powder. p. 10

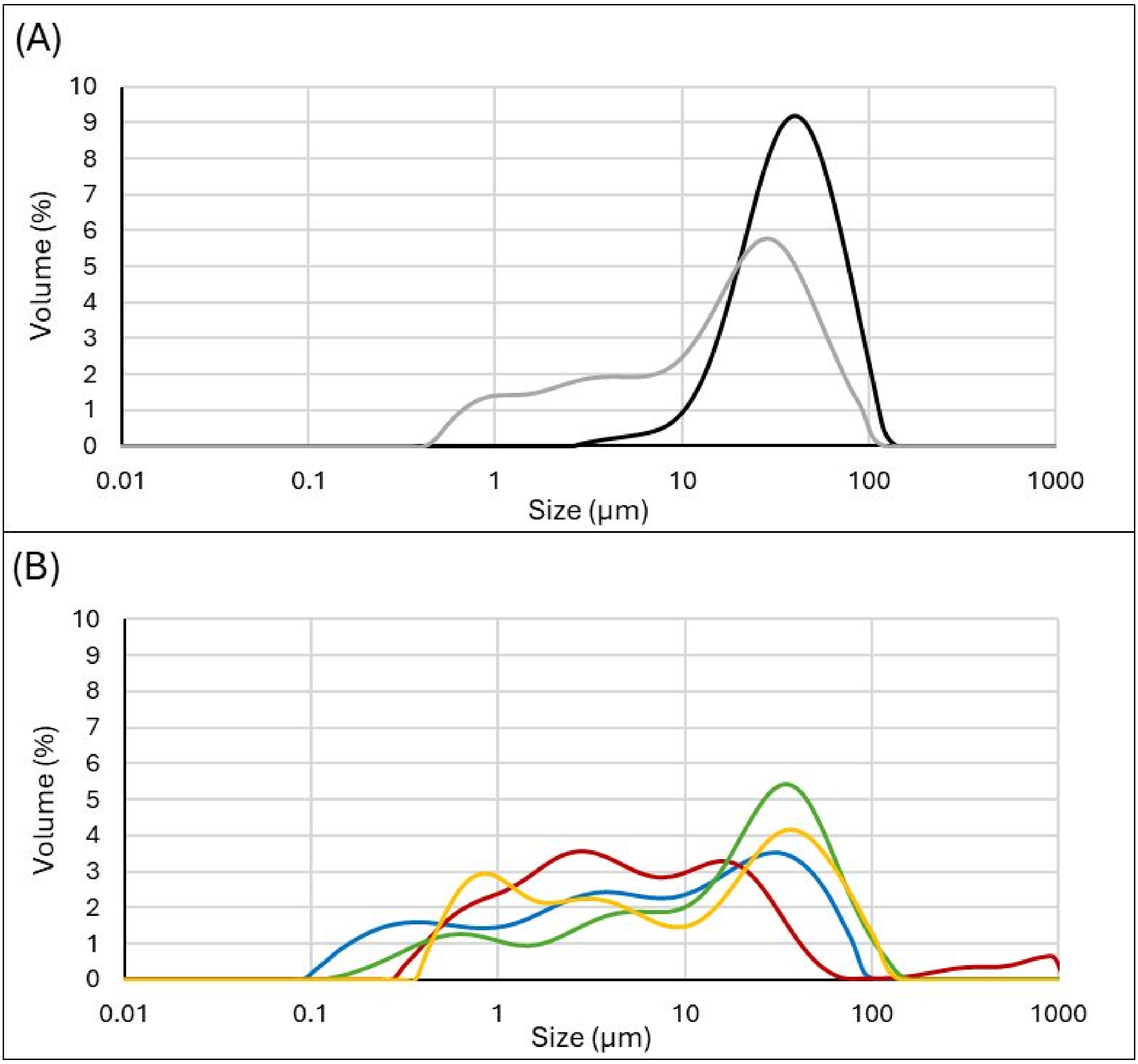

Figure S1. Particle size distribution profiles of the (A) starting materials (black line for FLG; grey line for oyster shell powder) and the (B) nanocomposites with different concentrations of FLG (yellow line for FLG 1%; green line for FLG 5%; red line for FLG 7%; blue line for FLG 10%).

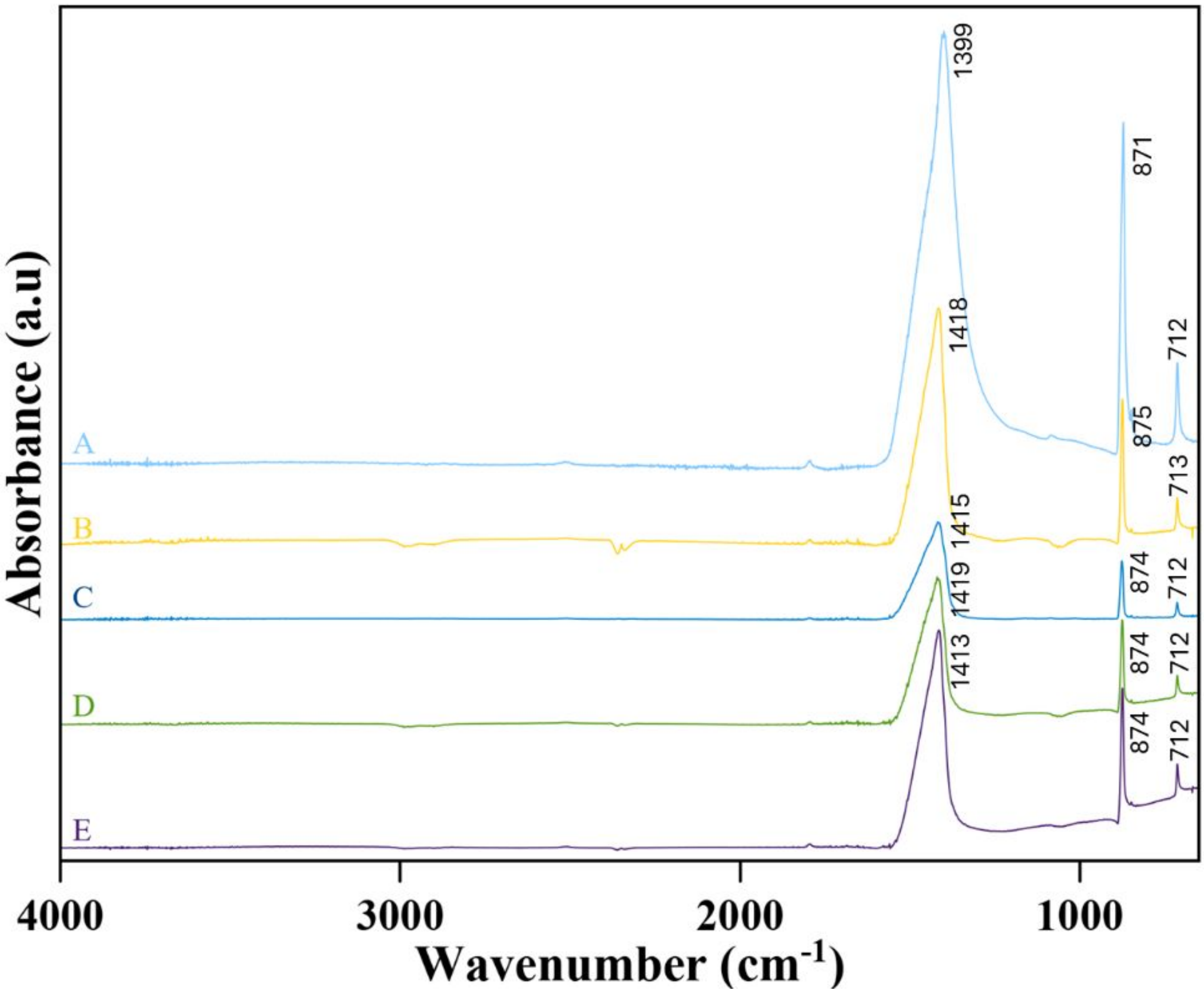

Figure S2. ATR-FTIR spectra of (A) oyster shell powder, (B) FLG 1%, (C) FLG 5%, (D) FLG 7%, and (E) FLG 10%.

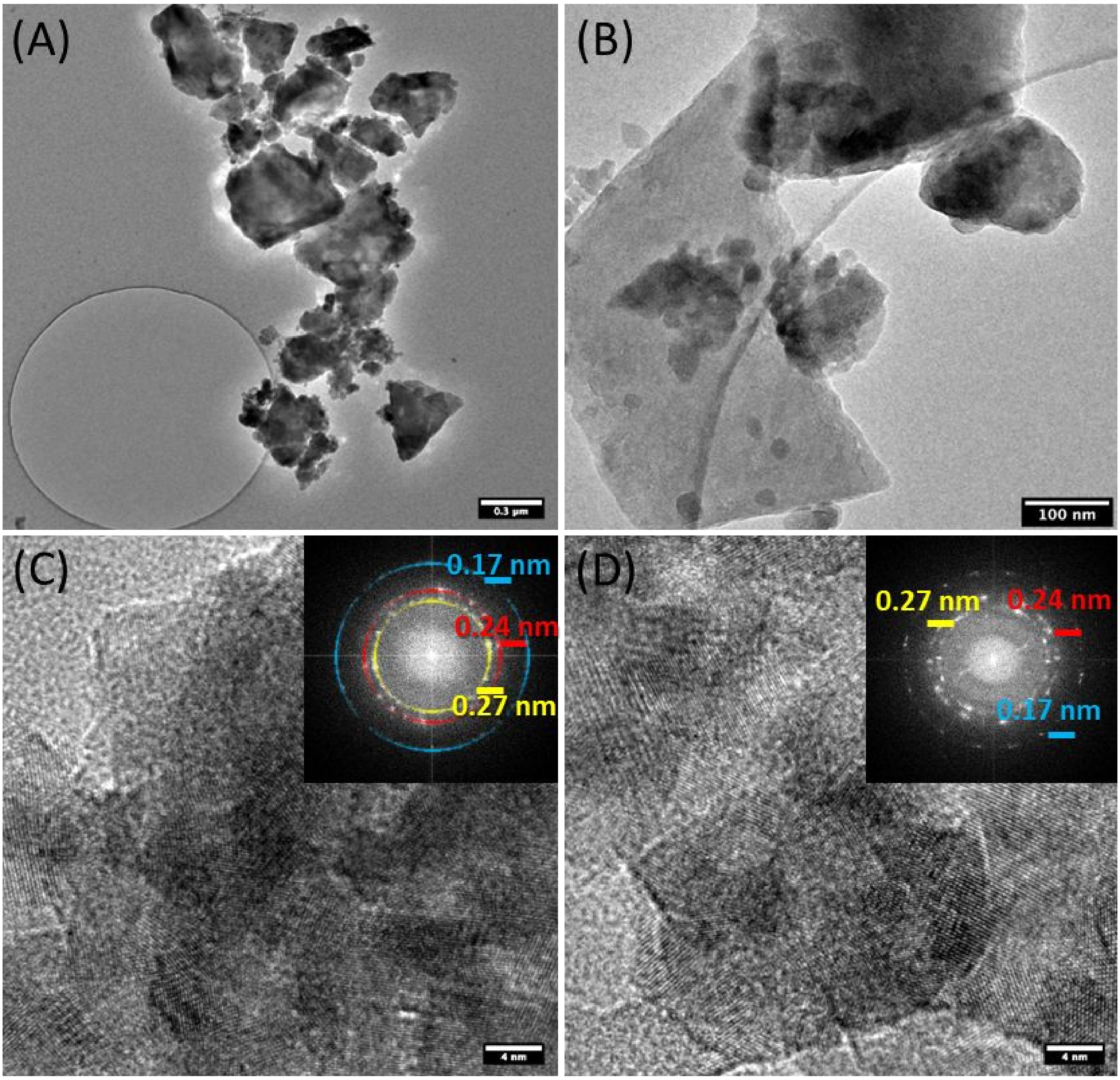

Figure S3. (A-D) TEM micrographs at different magnification of the oyster shell $CaCO_3$ powder. An insert on the top right corner of panel C shows the Fast Fourier transform (FFT) at high magnification where a polycrystalline pattern of calcite is observable.

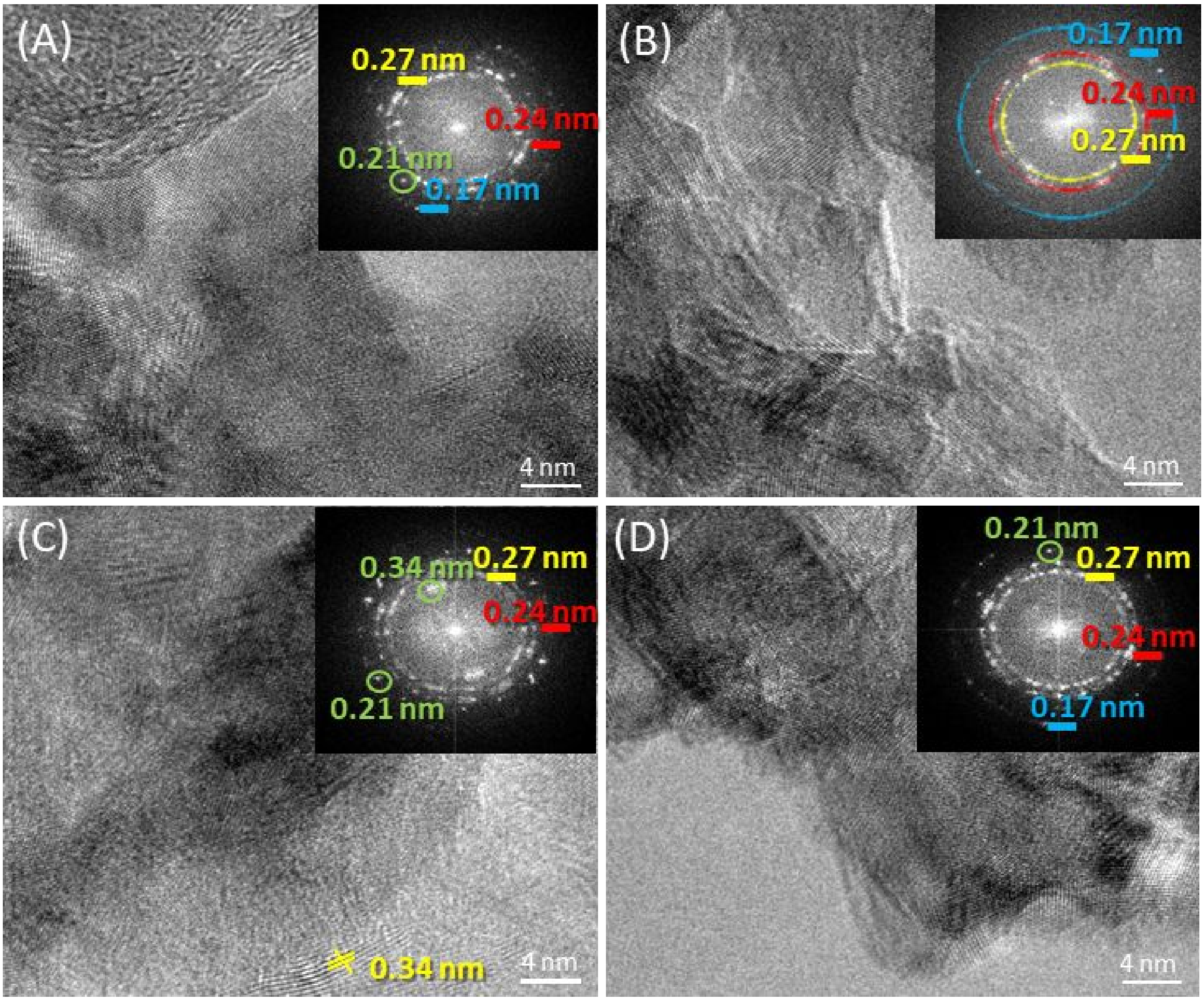

Figure S4. High-Resolution TEM of the (A) FLG 1%, (B) FLG 5%, (C) FLG 7%, and (D) FLG 10% with inset on the top-right corner of the image showing the Fast Fourier transform (FFT). In each FFT a polycrystalline diffraction pattern for calcite is observable (blue, red and yellow d-spacing) while on FLG 1%, FLG 7% and FLG 10% also the d-spacing of graphene are visible (green).

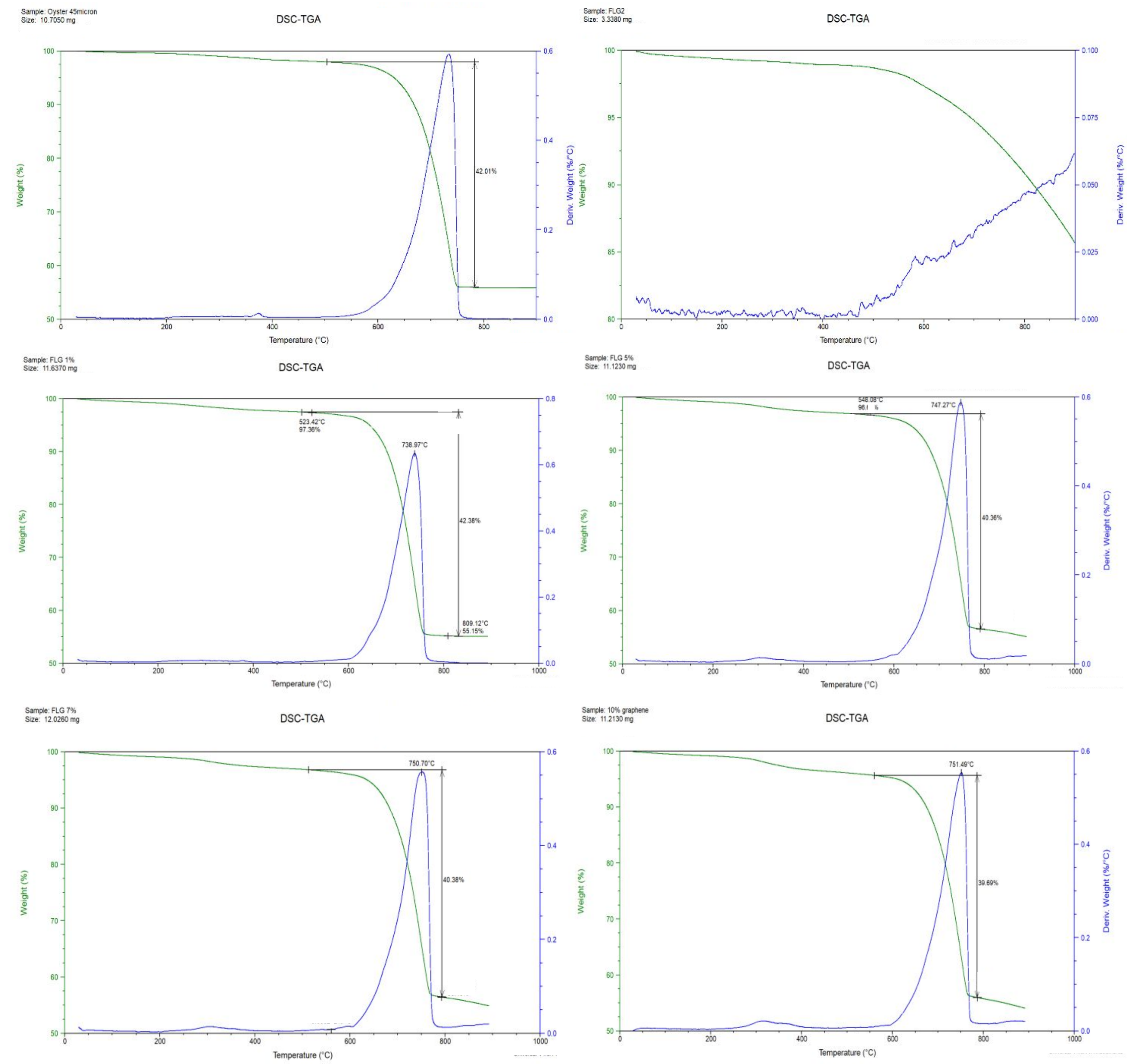

Figure S5. Thermogravimetric analysis (TGA) profiles with derivative of oyster shell powder, FLG, FLG 1%, FLG 5%, FLG 7%, FLG 10%.

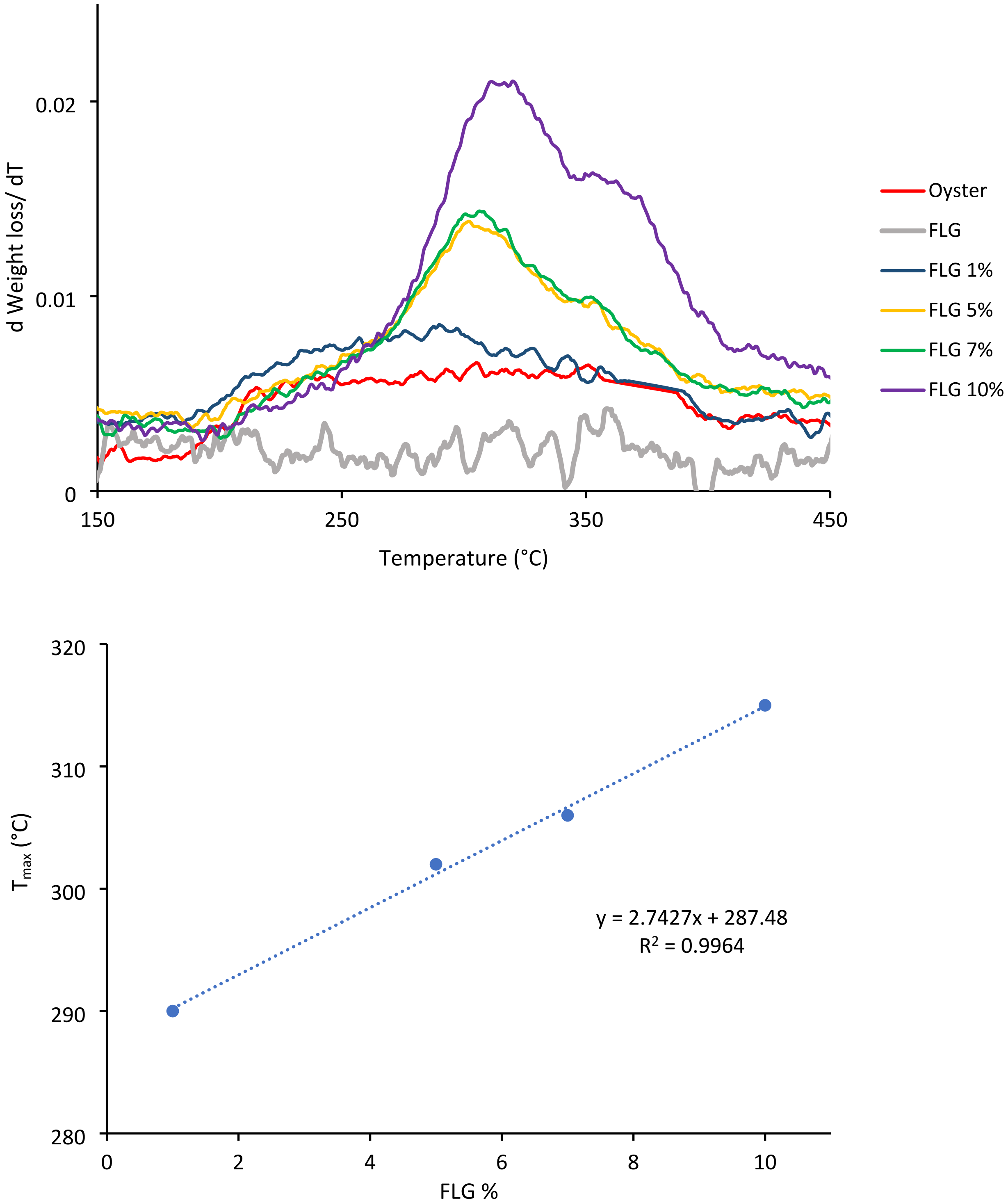

Figure S6. (above) Derivative of the weight loss signal over the temperature of the TGA profile in the range of the organic matrix degradation. (below) Linear trend of the temperature of maximum degradation of the organic matrix over the graphene content.

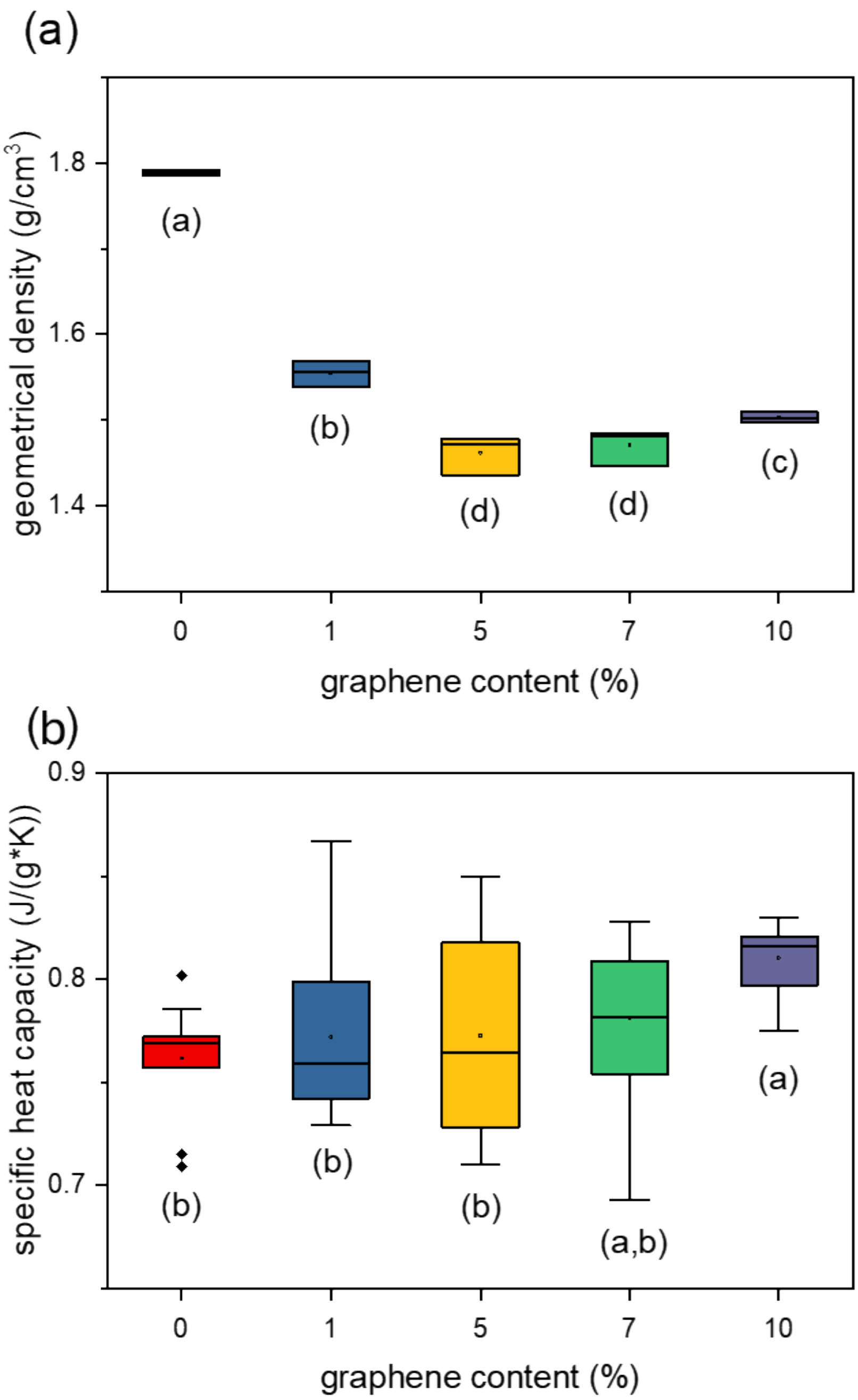

Figure S7. Boxplots of the geometrical density (a), and specific heat capacity (b) for the five analyzed specimens. We refer to the oyster powder as the sample reported with 0% graphene. Letters within each graphs represent the results of the Tukey's HSD test (same letter means no significant difference).

Table S1. List of the main diffraction peak angles of magnesium-calcite. Cell parameters were calculated from the listed Mg-calcite reflections using Bragg's law (Cu Kα, λ = 1.54056 Å) and a least-squares fit in the hexagonal setting (R-3c).

Sample	**2θ** (°)											**Unit-cell parameters**		
	(012)	(104)	(006)	(110)	(113)	(202)	(024)	(018)	(116)	(211)	(122)	**a** (Å)	**c** (Å)	**V** (Å³)
Oyster shell	23.10	29.43	31.45	36.01	39.44	43.20	47.16	47.52	48.52	56.60	57.44	4.9854 ± 0.0006	17.0554 ± 0.0042	367.10 ± 0.11
FLG1%	23.18	29.52	31.54	36.10	39.55	43.28	47.28	47.60	48.62	56.68	57.51	4.9768 ± 0.0018	17.0149 ± 0.0126	364.98 ± 0.32
FLG5%	23.25	29.59	31.57	36.19	39.62	43.38	47.37	47.67	48.70	56.80	57.61	4.9670 ± 0.0023	16.9942 ± 0.0162	363.10 ± 0.40
FLG7%	23.28	29.62	31.67	36.19	39.62	43.38	47.37	47.72	48.72	56.78	57.62	4.9679 ± 0.0027	16.9626 ± 0.0189	362.56 ± 0.47
FLG10%	23.33	29.70	31.60	36.24	39.68	43.42	47.49	47.75	48.76	56.89	57.66	4.9602 ± 0.0036	16.9645 ± 0.0247	361.47 ± 0.61

,

***Table S2**. Component balance for the 150–450 °C loss. Values are calculated assuming complete retention and complete P123 loss in the interval. OM concentrations are not directly measured.*

Formulation	Experimental loss (wt%)	P123 (wt%)	Calculated OM loss (wt%)
FLG 1%	1.72	0.062	1.484
FLG 5%	2.15	0.312	1.421
FLG 7%	2.11	0.436	1.389
FLG 10%	2.89	0.621	1.342

***Table S3**. Prospective uses of the shell-derived BCC/FLG powder. None of these applications is demonstrated in this study.*

Prospective use	Basis in the present results
Conductive or antistatic filler in a coating or polymer	Stable reported conduction in 5–10 wt% FLG pressed powders suggests possible charge-transport pathways.
Filler for moderate heat redistribution in polymer or mineral products	The 10 wt% compact reached 0.516 $W \cdot m^{-1} \cdot K^{-1}$ at 25 °C.
Conductive additive for cement or mortar, with sensing as a subsequent goal	A mineral-rich conductive powder could be evaluated for compatibility with cementitious matrices; related graphene/cement and GO/$CaCO_3$ studies provide context.[1,2]

1. Su, W.; Liu, C.; Bao, W.; Zheng, Z.; Ma, G.; Deng, Y.; Ye, W. Study on the Mechanical Performance, Durability, and Microscopic Mechanism of Cement Mortar Modified by a Composite of Graphene Oxide and Nano-Calcium Carbonate. Buildings 2024, 14 (7), 2236. https://doi.org/10.3390/buildings14072236
2. Jin, M.; Li, W.; Ma, Y.; Zeng, H.; Huang, M.; Lu, C.; Yang, G. Investigation on the Electrical Conductivity of Graphene/Cement Composites by Alternating Current Method. Materials 2023, 16, 1436. https://doi.org/10.3390/ma16041
